# Supplementary material for: Interface-acting nucleotide controls polymerization dynamics at microtubule plus- and minus-ends
Source: bioRxiv. 2023 Sep 27:2023.05.03.539131. Originally published 2023 May 4. Preprint. [Version 2] doi: 10.1101/2023.05.03.539131 (PMC10187237; doi:10.1101/2023.05.03.539131)
Supplement: Supplement 1 [file NIHPP2023.05.03.539131v2-supplement-1.pdf]

bioRxiv preprint doi: https://doi.org/10.1101/2023.05.03.539131; this version posted September 27, 2023. The copyright holder for this preprint (which was not certified by peer review) is the author/funder, who has granted bioRxiv a license to display the preprint in perpetuity. It is made available under aCC-BY 4.0 International license.

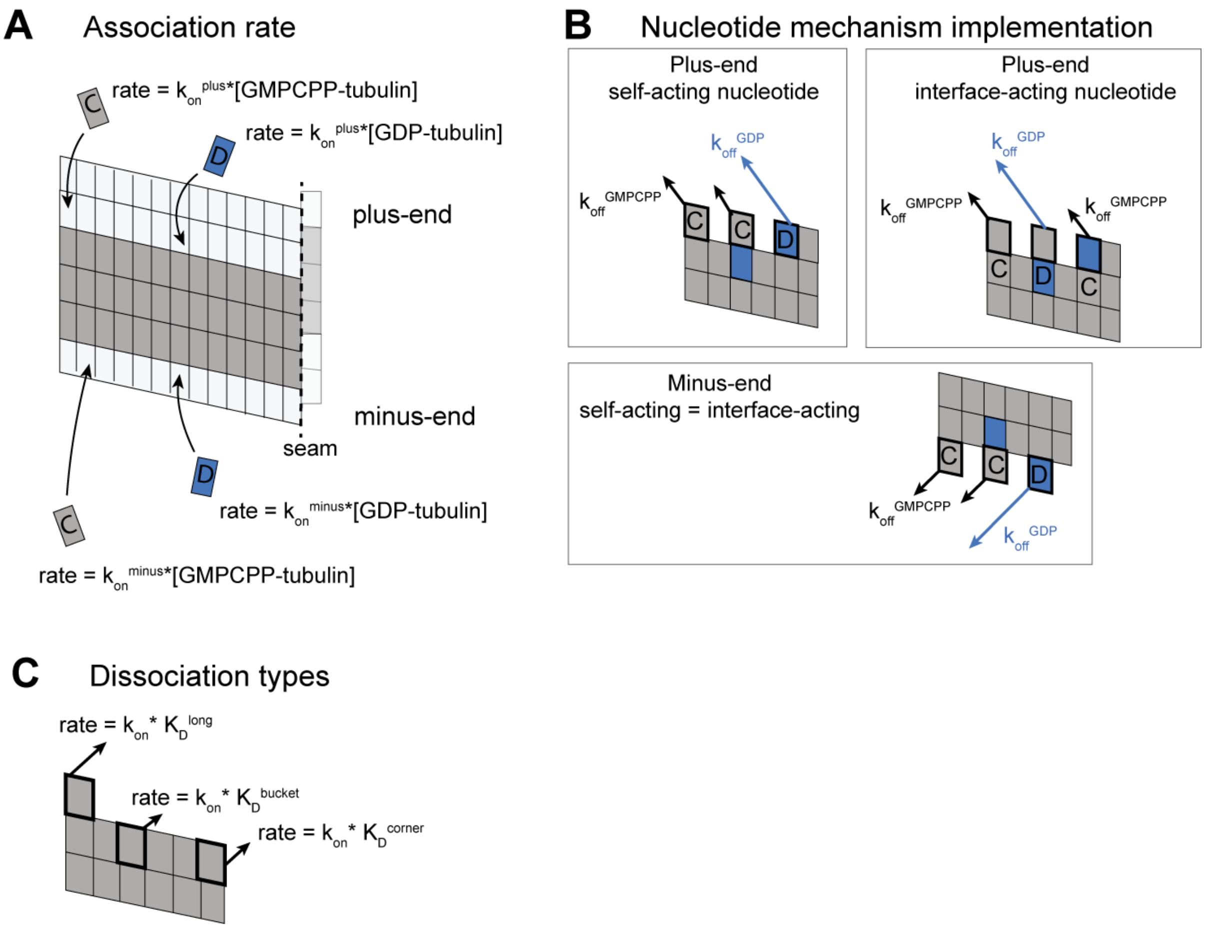

Figure 1 - figure supplement 1. Implementation of plus- and minus-end models. (A) The microtubule lattice is represented as a two-dimensional grid; interactions between edge protofilaments generate the seam (dashed line) and mimic the cylindrical nature of the microtubule lattice. Grey boxes represent GMPCPP-tubulin, blue boxes represent GDP-tubulin, and white boxes represent empty (nucleotide free) positions. Arrows show how new subunits can associate at the plus-end or minus-end, respectively. Simulations begin with a microtubule seed, shown here as three rows of GMPCPP-tubulin. Subunit on-rates are determined by the on-rate constant (k_{on}^{plus} or k_{on}^{minus}) and the concentration of tubulin. **(B)** Implementation of self-acting and interface-acting nucleotide mechanisms in plus-end simulations. Arrows indicate tubulin off-rates from the lattice, with black arrows denoting GMPCPP-tubulin off-rates and blue arrows denoting GDP-tubulin off-rates. **(C)** Tubulin dissociation rates from the lattice vary with the number of nearest neighbors. Plus-end simulations use the plus-end on-rate constant (k_{on}^{plus}), and minus-end simulations use the minus-end on-rate constant (k_{on}^{minus}).

bioRxiv preprint doi: https://doi.org/10.1101/2023.05.03.539131; this version posted September 27, 2023. The copyright holder for this preprint (which was not certified by peer review) is the author/funder, who has granted bioRxiv a license to display the preprint in perpetuity. It is made available under aCC-BY 4.0 International license.

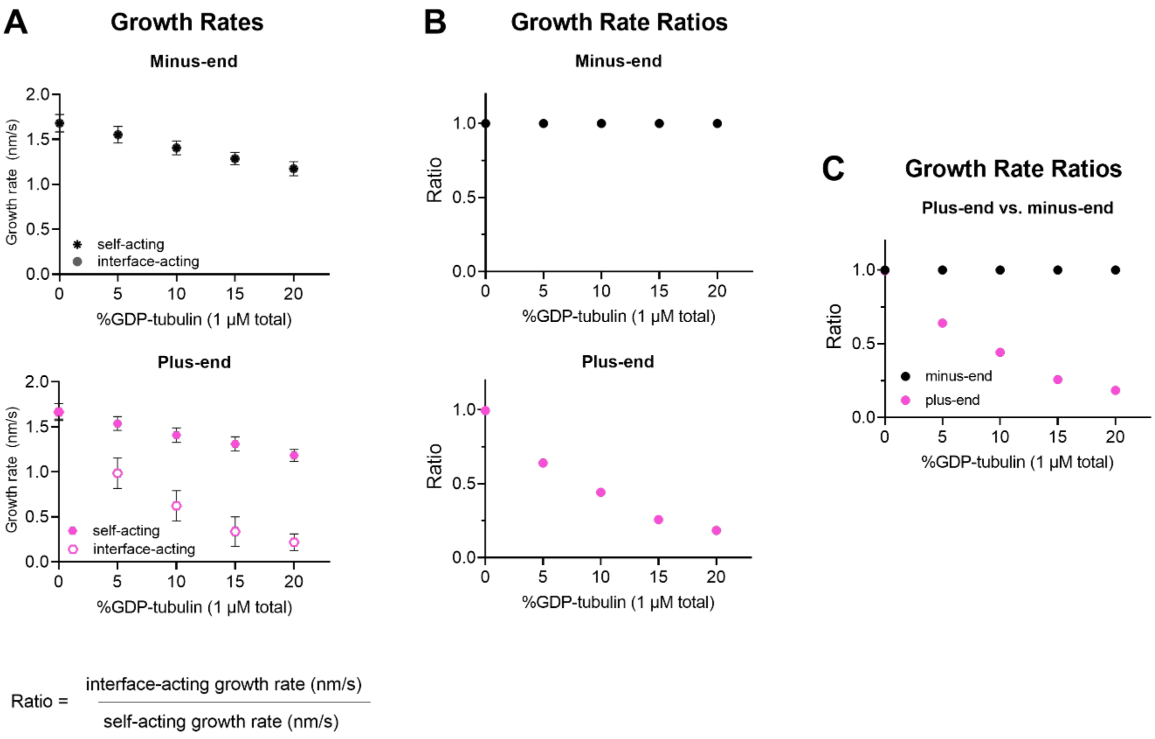

Figure 1 Supplement 2. Using simulated growth rates to predict differences between interface- and self-acting nucleotide mechanisms at plus-end and minus-ends. A) Simulated minus-end and plus-end growth rates (nm/s) for interface-acting or self-acting nucleotide mechanisms using parameters shown in Figure 1. If growth rate markers are not visible, they are obscured by another marker. Error bars are standard deviation, with n = 50 independent simulations per concentration of GDP-tubulin. **B)** Growth rate ratios are defined as the growth rate for the interface-acting mechanism divided by the growth rate for the self-acting nucleotide mechanism, as a function of the GDP-tubulin concentration. A ratio of 1 indicates that no difference in growth rates is predicted for self- and interface-acting nucleotide mechanisms. **C)** Growth rate ratios of plus- and minus-ends from panel **B)** plotted together to emphasize how self- and interface-acting mechanisms predict increasingly different plus-end growth rates with increasing GDP-tubulin, whereas the two mechanisms predict similar minus-end growth rates across a range of GDP-tubulin concentrations.

bioRxiv preprint doi: https://doi.org/10.1101/2023.05.03.539131; this version posted September 27, 2023. The copyright holder for this preprint (which was not certified by peer review) is the author/funder, who has granted bioRxiv a license to display the preprint in perpetuity. It is made available under aCC-BY 4.0 International license.

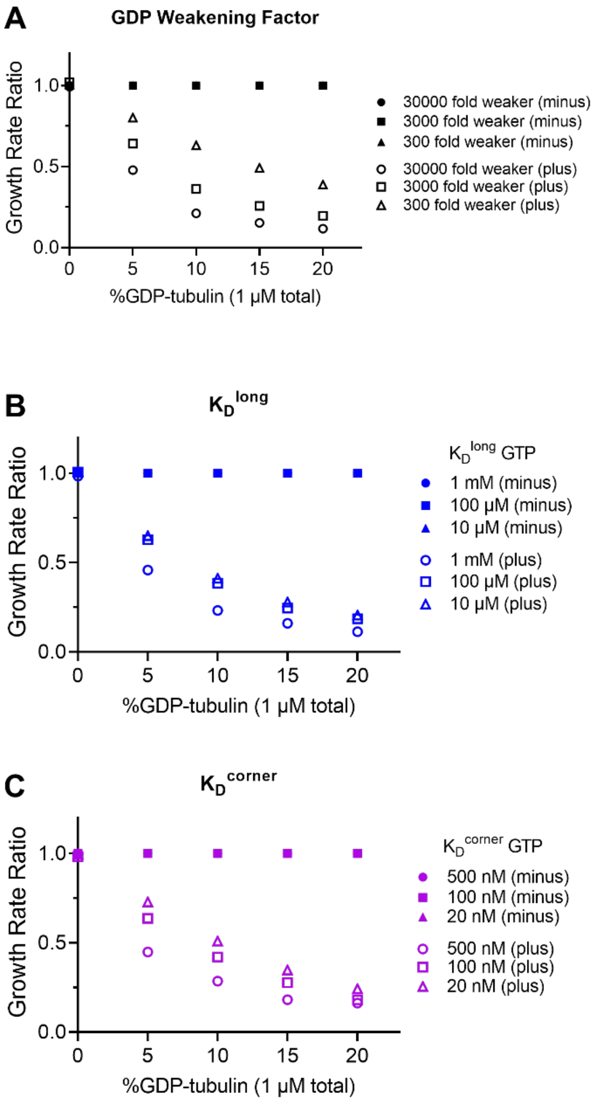

Figure 1 Supplement 3. Predicted differences between self- and interface-acting mechanisms at the plus-end are robust to variation in simulation parameters. A-C) Ratios of simulated plus-end (open symbols) and minus-end (filled symbols) growth rates for interface- and self-acting nucleotide mechanisms. Growth rate ratios are calculated by dividing the interface-acting growth rate by the self-acting growth rate. A ratio of 1 means that no difference in growth rates is predicted. For each simulation parameter, a weaker and stronger choice (relative to the value used in Figure 1) was tested. The predicted difference between interface-acting and self-acting mechanisms persists, even for different choices of **A)** GDP weakening factor (100-fold range), **B)** longitudinal interaction (K_D^{long}, 100-fold range), and **C)** corner interaction (K_D^{corner}, 25-fold range). The original conditions used in Figure 1 are: GDP weakening factor of 3000, K_D^{long} of 100 µM, and a K_D^{corner} of 100 nM. The GDP weakening factor describes the fold change between the GDP- and GTP-type interactions. N = 50 simulations per concentration.

bioRxiv preprint doi: https://doi.org/10.1101/2023.05.03.539131; this version posted September 27, 2023. The copyright holder for this preprint (which was not certified by peer review) is the author/funder, who has granted bioRxiv a license to display the preprint in perpetuity. It is made available under aCC-BY 4.0 International license.

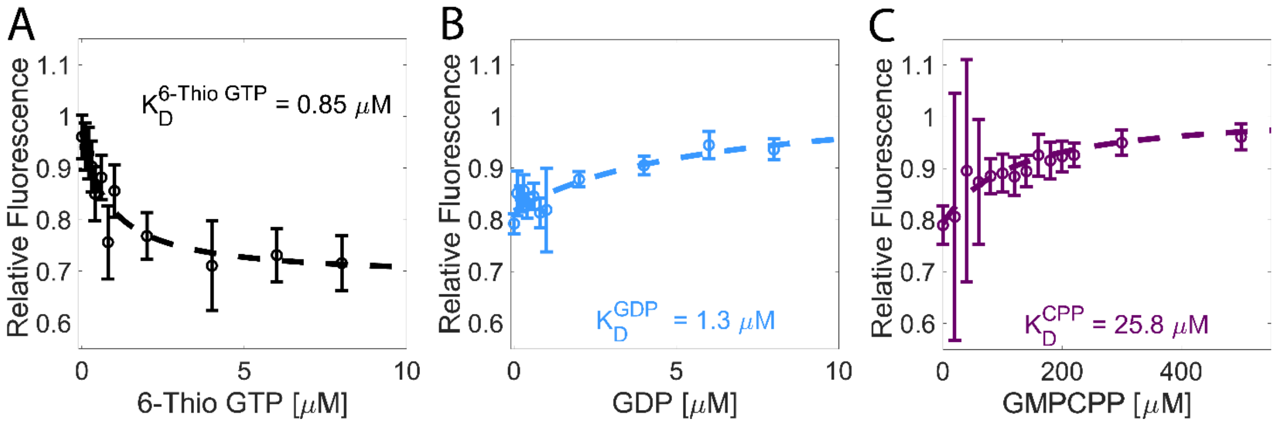

Figure 3 – figure supplement 1. **Tubulin has a higher affinity for GDP than for GMPCPP. (A)** The affinity of tubulin for 6-Thio GTP measured by nucleotide-dependent quenching of the tubulin tryptophan fluorescence. Values are the tubulin fluorescence (0.2 μM tubulin) divided by the fluorescence of a signal-matched BSA sample to correct for the inner filter effect. Error bars are SEM for n=6 determinations for each sample, accounting for errors in the concentrations of tubulin and BSA, and in the buffer control. **(B)** Determination of tubulin affinity for GDP. Increasing concentrations of GDP were added to a solution of 0.2 μM tubulin in the presence of 3 μM 6-Thio GTP. GDP displaces 6-Thio GTP from the tubulin, causing unquenching of tryptophan fluorescence. Error bars denote SEM with n=6-11 determinations per point. **(C)** Determination of tubulin affinity for GMPCPP using an identical approach. Error bars are SEM with n=6 determinations per point.

bioRxiv preprint doi: https://doi.org/10.1101/2023.05.03.539131; this version posted September 27, 2023. The copyright holder for this preprint (which was not certified by peer review) is the author/funder, who has granted bioRxiv a license to display the preprint in perpetuity. It is made available under aCC-BY 4.0 International license.

A Nucleotide exchange

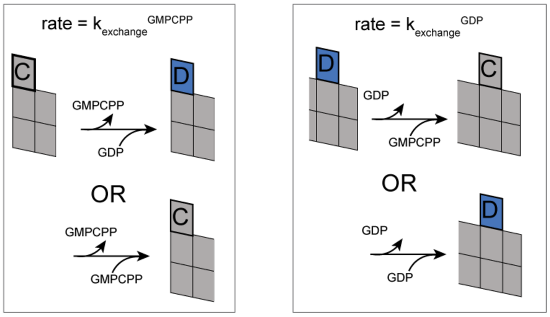

probability of replacement with GDP or GMPCPP depends upon concentration of either nucleotide

B

GDP Exchange Rate	RMSE (Root Mean Squared Error)	Fraction Total Error (at 0 s^{-1})
0 s^{-1}	0.59	1
0.01 s^{-1}	0.42	0.71
0.05 s^{-1}	0.21	0.35
0.1 s^{-1}	0.11	0.19
0.3 s^{-1}	0.05	0.08
0.5 s^{-1}	0.03	0.06
0.7 s^{-1}	0.05	0.08
0.9 s^{-1}	0.05	0.08

Figure 5 – figure supplement 1. Implementation and analysis of nucleotide exchange (A) Implementation of nucleotide exchange in simulations of plus-ended growth. Terminal exposed subunits can undergo exchange with a finite first-order rate, $k_{exchange}$ (s^{-1}). The probability of exchange to GDP or GMPCPP is determined by the relative concentration of each nucleotide. **(B)** Root-mean-squared error (RMSE) of predicted growth rates vs. experimental growth rates for a series of exchange rates, across the tested range of GDP- and GMPCPP-tubulin mixtures. Fraction total error is defined as the relative error compared to the error when exchange rate is 0 s^{-1}.
